# Modulation of nociceptive threshold by combined hormonal contraceptives in women with oestrogen-withdrawal migraine attacks: a pilot study

**DOI:** 10.1186/s10194-016-0661-6

**Published:** 2016-08-04

**Authors:** Roberto De Icco, Laura Cucinella, Irene De Paoli, Silvia Martella, Grazia Sances, Vito Bitetto, Giorgio Sandrini, Giuseppe Nappi, Cristina Tassorelli, Rossella E. Nappi

**Affiliations:** 1 Headache Science Centre, C. Mondino National Neurological Institute, University of Pavia, Pavia, Italy; 2 Department of Brain and Behavioral Sciences, University of Pavia, Pavia, Italy; 3 Research Centre for Reproductive Medicine, Gynaecological Endocrinology and Menopause, IRCCS San Matteo Foundation, Pavia, Italy; 4 Department of Clinical, Surgical, Diagnostic and Paediatric Sciences, University of Pavia, Pavia, Italy

**Keywords:** Oestrogen withdrawal headache, Menstrually related migraine, Combined hormonal contraceptives, Nociceptive withdrawal reflex

## Abstract

**Background:**

Menstrually-related headache and headaches associated with oestrogen withdrawal are common conditions, whose pathophysiology has not been completely elucidated. In this study we evaluated the influence of combined hormonal contraceptives (CHC) on pain threshold in women presenting migraine attacks during hormone-free interval.

**Findings:**

Eleven women with migraine attacks recurring exclusively during the oestrogen-withdrawal period were studied with the nociceptive flexion reflex, a neurophysiological assessment of the pain control systems, during the third week of active treatment and during the hormone-free interval.

During the hormone-free interval, nociceptive withdrawal reflex threshold was significantly lower (12.8 ± 8.0 mA) as compared to the third week of hormonal treatment (15.6 ± 6.6 mA) (*p* = 0.02). No change was observed in the pain perceived and in the temporal summation.

**Conclusions:**

Oestrogen withdrawal may mediate an increased sensitivity to somatosensory stimuli in women with migraine attacks recurring during the hormone-free interval.

## Introduction

Migraine is a common and disabling condition mostly affecting women during fertile age; reproductive milestones, as well as hormonal fluctuations across menstrual cycle and exogenous hormonal interventions hugely influence migraine clinical course in women. In observational studies, up to 60 % of migrainous women reported an association between menstruation and migraine attacks, often described as more severe, longer lasting and more resistant to treatment than non-menstrual ones [1]. Since a long time, oestrogen fall during late luteal phase has been implicated as a major event triggering migraine attacks [2]; oestrogen withdrawal has also been reported to induce headache in non migrainous patients [3]; this clinical entity has been codified in the International Headache Disorders Classifications (ICHD) as oestrogen-withdrawal hedache [4].

Events following oestrogens drop and predisposing to headache remain to be fully elucidated. Among the others, modulation of pain control system by sex hormones has been suggested to play a role: in a previous study, we assessed pain threshold in healthy women during the follicular and luteal phases of spontaneous cycles by investigating the threshold for nociceptive withdrawal reflex triggered by electrical stimulation of the sural nerve [5]. We observed that the pain threshold was significantly lower during the luteal phase and this finding was associated with an increased perception of physical disturbances, as assessed through the menstrual distress questionnaire. This is likely to result from a complex interaction between neurotransmitters and sex steroids, whose fluctuations however cannot be easily predicted in spontaneously menstruating women. That being so, combined hormonal contraception (CHC) represents a good model to study the “menstrual window” of vulnerability, because during the hormone-free interval the fluctuations of exogenous hormones are sizeable and predictable. Even though modern hormonal contraception encompasses multiple choices, the most commonly used is “the pill” in which the hormone free interval lasts seven days (21/7 regimens) and CHCs are discontinued to obtain withdrawal menstrual bleeding following 21 days of use. The same is true for the patch and the ring, which contain as well a combination of estrogen and progestogen [6]. Because of the steep decline of ethinyl oestradiol levels, ranging usually from 15 to 30 μg, the so-called oestrogen withdrawal symptoms may occur and likely prevented by extended CHC regimens to avoid the hormone-free interval [7].

In order to further explore the relation between oestrogen-withdrawal and headache, in this study we evaluated changes in the nociceptive withdrawal reflex threshold in women using CHC and reporting migraine attacks consistently and exclusively in the hormone-free interval.

## Methods

We enrolled in a pilot study 11 women who were using CHC with a 21/7 pattern and who reported migraine attacks that occurred exclusively during the hormone-free interval of CHC in the previous 3 months. Patients fulfilling the inclusion criteria were consecutively enrolled among those attending the outpatient clinic of the Headache Science Center of the “C. Mondino” National Neurological Institute. The CHC formulation was oral (20 or 30 mcg ethynil estradiol pill) in 5 patients, transdermal (20 mcg ethynil estradiol patch) in 2 patients and vaginal (15 mcg ethynil estradiol ring) in 4 patients. All the patients followed the same therapeutic cycle with 21 days of active treatment followed by a seven-day break (the hormone-free interval) during which withdrawal (scheduled) bleeding occurred.

Their mean age was 30.2 ± 8.4 years (range 19–42 years). An exploratory analysis was also performed according to the onset of headache with respect to CHC treatment: in 5 women (mean age 30.6 ± 8.2 years) a headache that fulfilled criteria for migraine without aura was present prior to the exposure to CHC, while in the other 6 women the onset of headache occurred in strict temporal association with CHC use. It is noteworthy that according to ICHD-III criteria beta version, patients in the first subset received a double diagnosis (1.1 migraine without aura + 8.3.3 Oestrogen withdrawal headache), while patients in the second subset were diagnosed as 8.3.3 Oestrogen withdrawal headache.

Each subject was assessed twice across the same CHC cycle: during the third week of active treatment (T0) and during the hormone free interval (T1); all women were headache-free at the time of testing. Nociceptive withdrawal reflex was evaluated at the lower limb level, through electrical stimuli delivered percutaneously at the sural nerve; the muscular response was recorded from the biceps femoris using an electromyografic technique. The reflex threshold following a single stimulus (RT-SS) was considered as the lowest current intensity needed to elicit a stable electromyografic response (mA) (see ref. [8]). The area under the curve was also calculated (μV*ms), as an estimate of the motor units recruited upon reflex trigger. Subjective pain intensity perceived by the patient during the delivery of stimuli with an intensity corresponding to RT-SS was rated by participants on an 11-point scale (Visual Analogue Scale, VAS), ranging from 0 = no pain to 10 = unbearable pain.

Temporal summation of the nociceptive flexion reflex (RT-TS), which represents a reliable objective measure of the functional activity of the nociceptive system in the spinal cord, was also evaluated using train of 5 stimuli at a frequency of 2 Hz. It was defined as the lower stimulation intensity (mA) generating a clear facilitation of the reflex response size (greater than 20 µV for 10 ms or more) in the fourth and fifth trace during the course of the five individual pulses train (for details see ref. [9]).

## Findings

**Fig. 1:**
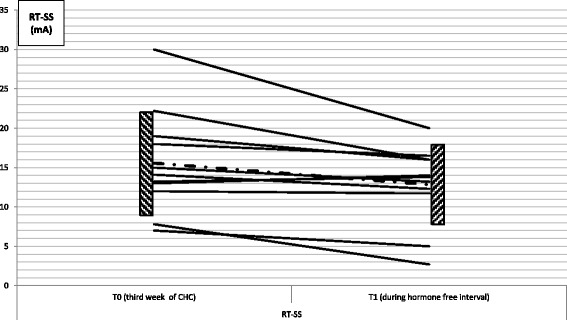
Changes in the threshold of the nociceptive flexion reflex following a single stimulus (RT-SS) at T0 and T1 in our population. Dashed line and dashed boxes illustrate the mean ± standard deviation of the reflex. Student’s t test for paired data (T1 vs T0, *p* = 0.02). Y axis: intensity of the stimulation at RT-SS in mA

During the third week of CHC (T0), the mean RT-SS was 15.6 ± 6.6 mA, with an average area under the EMG track of 1287.9 ± 738.1 μV*mS, while during hormone free interval (T1), mean RT-SS was found to be significantly lower at 12.8 ± 8.0 mA (T1 vs T0, *p* = 0.02) (Fig. 1), with a tendency toward an increase in the reflex area, which however did not reach a statistically significant level (*p* = 0.158). No change was observed in terms of subjective pain perception at the reflex threshold as reported by the patient through VAS (RT-VAS at T0 = 5.41 ± 2.08; RT-VAS at T1 = 5.41 ± 2.02; *p* = 1.00). No differences were seen as regards temporal summation recorded at T0 and at T1.

**Fig. 2:**
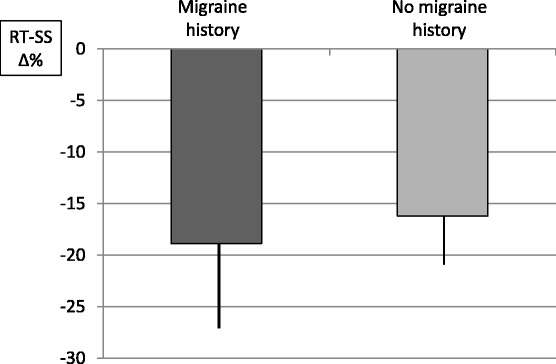
Percent reduction in the nociceptive flexion reflex threshold during the suspension week in women with a positive history of migraine (left column) and in women with a negative history of migraine attacks before the use of combined hormonal contraceptives (CHC) (right column). Data are presented as mean ± standard error. Student’s t test for paired data, *p* > 0.05. Y axis: percentage reduction of RT-SS (RT-SS Δ%) during hormone-free interval respect to baseline

As far as the exploratory analysis is concerned, the drop in reflex threshold at T1 with respect to T0 was similar in the 2 subgroups of women: migraine onset before CHC Δ RT-SS = −18.90 ± 12.66; migraine onset after CHC Δ RT- SS = −16.18 ± 29.31) (Fig. 2).

## Discussion

Our findings indicate that the hormone-free interval of CHC is associated to a reduction in the RT-SS of the lower limb nociceptive reflex in women with attacks of migraine induced by oestrogen-withdrawal. No change in subjective pain intensity was perceived at RT-SS from T0 to T1, meaning that during hormone free interval, participants rated an electrical stimulus at lower current intensity as painful as an electrical stimulus at higher current intensity necessary to elicit the nociceptive withdrawal reflex during the third week of active treatment. Thus, the objective lowering of neurophysiologically recorded RT-SS during hormone-free intervals was indeed coupled to an increase in the subjective self-reported pain sensitivity.

Although not statistically significant, likely due to the paucity of the study sample, we found a trend towards an increase in the area under the curve at RT-SS during the withdrawal week. This observation suggests that during the hormone-free interval more motor units can be recruited at RT-SS intensity. If this result is confirmed in a larger sample of patients, it would strongly suggest a role for oestrogen withdrawal in the NWR facilitation at the spinal level.

It is noteworthy that we observed the same pattern of neurophysiological and subjective responses in two different subgroups from a nosographic point of view: 1) migraineurs whose pre-existing headache was markedly modified by CHC as regards the temporal pattern of occurrence of attacks and 2) healthy women whose headache is secondary to the use of CHC. Though obtained in a small sample, this finding prompts the hypothesis that the observed neurophysiological similarities between these 2 subgroups do reflect a shared pathophysiological alteration of the nociceptive network mediated by the drop in oestrogen levels. In this regard, it is noteworthy that the working methodology in this study was to enroll patients who shared the same migrainous phenotype of attacks together with the same temporal pattern of their occurrence.

We are aware that our study suffers some limitations mainly represented by the small sample of patients and the lack of a control group. We tried indeed to limit the impact of these biases with a strict selection of patients and with the choice of a validated neurophysiological methodology to assess pain threshold.

Moreover, the main purpose was to validate and extend our previous observation of a reduction of the RT-SS in healthy women during the luteal phase [5]. Taken together, these data suggest that the decrease in oestrogen levels makes women more ‘receptive’ to pain as a default response. The predisposition to develop headache or migraine attacks could be related to events downstream of the oestrogen drop and that remain mostly elusive. Previous studies attempted to investigate the influence of hormonal contraceptives on pain threshold, with conflicting results [10, 11], probably because of methodological differences. Teepker et al. [10] in particular evaluated the pain thresholds of different types of stimuli (heat, cold, pressure and electrical) and found no difference between patients with or without CHC. In agreement with our results, other authors described fluctuations electrical pain thresholds along the menstrual cycle [11]. A recent study that evaluated the effect of hormonal contraception on pain sensitivity showed that users of progestin only contraceptive have higher pressure pain thresholds than CHC users or non-users, thus suggesting that also progestin do play a role, besides oestrogens, in the modulation of pain control systems [12].

The association between migraine and oestrogens has been widely studied and recently Chai et al. wrote a comprehensive review on this issue [13]. In particular, data on the effect of exogenous sex hormones showed that oestrogens are able to modify headache patterns in patients with migraine and that the avoidance of a marked drop of oestrogen levels in the blood may beneficial in preventing migraine occurring during estrogen withdrawal.

Different hypotheses have been proposed to explain the association between migraine attacks and hormonal fluctuation. According to the “mismatch theory” [14], a sudden oestrogen drop would result in a transient disequilibrium between genomic and non-genomic oestrogen-mediated actions on neurotransmission, vascular reactivity, neuroinflammation and other systems, overall promoting neuronal activation. Among the others, oestrogen modulation of serotonergic system seems to play a major role in influencing pain control system: a previous study conducted in women presenting *status migrainosus* during the hormone free interval of CHC, showed a blunted cortisol and prolactin secretion, following a serotonergic challenge (M-phenylchloropiperazine); moreover, the status migrainosus could be prevented by transdermal estradiol supplementation against placebo [15].

## Conclusions

In this pilot study we described a reduction of neurophysiologically recorded pain threshold in women with migraine/headache attacks during the hormone-free interval of CHC. The drop in oestrogen levels is likely to play a role in modulating nociception during the hormone-free interval. Further investigations in the neurobiological events leading to these attacks are necessary for understanding the biological aspects of CHC-induced attacks. The observation of the same pattern in women with or without a positive history of migraine could be important from both a nosographic and a therapeutic point of view.

## Abbreviation

CHC, combined hormonal contraceptives; RT-SS, nociceptive withdrawal reflex threshold elicited with a single stimulus; RT-TS, nociceptive withdrawal reflex threshold elicited with temporal summation; VAS, visual analogue scale

## References

[1] Granella F, Sances G, Allais G (2004). Characteristics of menstrual and non menstrual attacks in women with menstrually-related migraine referred to headache centers. Cephalalgia.

[2] Somerville BW (1975). Estrogen withdrawal migraine. Neurology.

[3] MacGregor EA (2013). Contraception and headache. Headache.

[4] Headache International Subcommittee of the International Headache Society (2013). International classification of headache disorders, 3rd edition (beta version). Cephalalgia.

[5] Tassorelli C, Sandrini G, Cecchini AP (2002). Changes in nociceptive flexion reflex threshold across the menstrual cycle in healthy women. Psychosom Med.

[6] Bitzer J, Simon JA (2011). Current issues and available options in combined hormonal contraception. Contraception.

[7] Sulak PJ, Kuehl TJ, Ortiz M (2002). Acceptance of altering the standard 21-day/7-day oral contraceptive regimen to delay menses and reduce hormone withdrawal symptoms. Am J Obstet Gynecol.

[8] Sandrini G, Rossi P, Milanov I, Serrao M, Cecchini AP, Nappi G (2006). Abnormal modulatory influence of diffuse noxious inhibitory controls in migraine and chronic tension-type headache patients. Cephalalgia.

[9] Perrotta A, Bolla M, Anastasio MG, Serrao M, Sandrini G, Pierelli F (2016). Modulation of temporal summation threshold of the nociceptive withdrawal reflex by transcutaneous spinal direct current stimulation in humans. Clin Neurophysiol.

[10] Teepker M, Peters M, Kundermann B (2011). The effects of oral contraceptives on detection and pain thresholds as well as headache intensity during menstrual cycle in migraine. Headache.

[11] Isselée H, De Laat A, Bogaerts K (2001). Long-term fluctuations of pressure pain thresholds in healthy men, normally menstruating women and oral contraceptive users. Eur J Pain.

[12] Máximo MM, Silva PS, Vieira CS (2015). Low-dose progestin releasing contraceptives are associated with a higher pain threshold in healthy women. Fertil Steril.

[13] Chai NC, Peterlina BL, Calhounb AH (2014). Migraine and estrogen. Curr Opin Neurol.

[14] Welch KM, Brandes JL, Berman NE (2006). Mismatch in how oestrogen modulates molecular and neuronal function may explain menstrual migraine. Neurol Sci.

[15] Nappi RE, Sances G, Brundu B (2005). Estradiol supplementation modulates neuroendocrine response to M-chlorophenylpiperazine in menstrual status migrainosus triggered by oral contraception-free interval. Hum Reprod.

